# Perfectionism as a moderator of the relationship between orthorexia nervosa and obsessive–compulsive symptoms

**DOI:** 10.1007/s40519-023-01629-1

**Published:** 2024-01-10

**Authors:** Phillipa Ann Huynh, Stephanie Miles, Maja Nedeljkovic

**Affiliations:** 1 Centre for Mental Health and Brain Sciences, Swinburne University of Technology, Hawthorn Campus, PO Box 218, Hawthorn, VIC 3122 Australia; 2 Centre for Youth Mental Health, The University of Melbourne, Melbourne, Australia; 3 Orygen, Melbourne, VIC Australia; 4 Department of Psychological Sciences, Swinburne University of Technology, Melbourne, VIC Australia

**Keywords:** Orthorexia nervosa, Obsessive–compulsive, Perfectionism, Healthy eating, Eating disorders

## Abstract

**Purpose:**

Orthorexia nervosa (ON), a proposed disorder describing an obsessive focus on “healthy” eating, is characterised as having overlapping symptoms with obsessive–compulsive disorder. However, ON/obsessive–compulsive (OC) symptom relationships are inconsistently reported. The current study aimed to investigate if the contribution of OC symptoms and beliefs explain variability in ON symptoms and determine if perfectionism, a transdiagnostic factor, moderates the ON/OC symptom relationship.

**Methods:**

The study comprised 190 participants (*M*_age_ = 28.63, *SD*_age_ = 9.88; 80% female) recruited via an undergraduate research programme, social media, advocacy organisations, and a participant registry. Participants completed an online questionnaire assessing ON, OC, and perfectionism symptoms.

**Results:**

A linear regression analysis found OC symptoms and beliefs explained 22.9% variability in ON symptoms (*p* < 0.001, *f*^2^ = 0.38) and perfectionism moderated the ON/OC symptom relationship, where higher levels of perfectionism with higher levels of OC symptoms was associated with higher levels of ON symptoms, explaining 2.2% variability (*p* = .01, *f*^2^ = 0.03).

**Conclusion:**

OC symptoms appear more common in ON than previous studies indicate. However, the interaction between perfectionism and OC symptoms may drive obsessions in ON. Findings help refine our current understanding of ON phenomenology with implications for ON treatment development. Future research should further explore perfectionism in ON phenomenology.

*Level of evidence***:** Level V (Opinions of respected authorities, based on descriptive studies, narrative reviews, clinical experience, or reports of expert committees).

## Introduction

Orthorexia nervosa (ON) is a proposed mental illness characterised by a fixation on eating “healthy” foods, resulting in psychological, physical, and/or social impairment [1]. Similar to the eating disorder (ED) anorexia nervosa, individuals with orthorexic tendencies rigidly restrict their food intake in line with rules surrounding “health” [2]. ON symptoms also resemble characteristics of obsessive–compulsive disorder (OCD) [2]. Similar to OCD, individuals with ON appear to experience repetitive thoughts and/or impulses and demonstrate compulsive behaviours to prevent a feared outcome, centred around health and “purity” of foods [2, 3]. ON research has predominantly investigated disordered eating symptoms with less attention given to obsessive–compulsive (OC) symptoms [4]. Further, clinically significant perfectionism, where pursuing self-imposed demanding standards regardless of negative consequences, has been associated with both ED and OC symptoms [5]. Understanding how OC symptoms present in ON may allow more targeted ON treatment interventions and assist in ON phenomenological research.

ON phenomenology is still being investigated and only recently has consensus diagnostic criteria been established in the literature after several attempts [1, 6–9]. ON is not recognised as a disorder in the *Diagnostic and Statistical Manual of Mental Disorders* (DSM-5-TR) [10] or the *International Statistical Classification of Diseases and Related Health Problems* (ICD) [11], contributing further to the ambiguity of ON’s definition. Dunn and Bratman characterised ON as an obsessive focus on “healthy” eating (irrespective of the belief and/or theory informing what the individual defines as “healthy”) and compulsively following a rigid diet [1]. As such, consuming “unhealthy” foods is emotionally distressing and often associated with a fear of disease, shame, and/or anxiety [1]. Subsequently, individuals demonstrating orthorexic tendencies can become socially isolated, avoiding situations where they are unable to control food quality [12, 13]. Further, individuals can eliminate entire food groups resulting in malnutrition [6]. These co-occurring symptoms of malnutrition, potential weight loss, and food restriction inform the comparison of ON to disorders such as anorexia nervosa.

### Empirically overlapping ON, ED, and OCD symptoms

ON has many similarities with EDs, including a preoccupation with food, needing a sense of control, and experiencing guilt after dietary transgressions [12, 14–16]. Further, symptom overlap between ON and anorexia nervosa includes ego-syntonic behaviours, where behaviours are congruent with ideal self-image, perfectionism, and strict food restrictions [14]. However, weight loss and a fear of becoming fat are typically motivating factors for anorexia nervosa and bulimia nervosa, whereas ON motivation appears to stem from a fear of being impure, unhealthy, or succumbing to disease [17, 18], although some research in a university sample found weight loss can also be a motivator in ON [19].

EDs are comorbid with obsessive–compulsive and related disorders [20] and several OC symptoms overlap with ON [14]. Similar to OCD, ON symptoms include perseverative thoughts about health and/or food quality, recurrent intrusive thoughts and fears around potential disease, and concerns over food contamination (e.g., from pesticides and/or genetically modified organisms). These overlapping symptoms all potentially cause intense anxiety and/or distress [17, 18, 21]. Further, in ON, food preparation can be ritualistic, including opting for natural cookware and avoiding aluminium in fear of contamination or poisoning, as is observed in OCD [15, 17, 22].

Unlike the ego-syntonic behaviour observed in ON, OCD behaviours tend to be ego-dystonic, or incongruent with ideal self-image, although some evidence suggests contamination fears are ego-syntonic [23]. Further, unlike ON, obsessions are not voluntary or pleasurable for people with OCD [2, 3]. Additionally, obsessions and compulsions of OCD typically extend beyond food and health, whereas in ON obsessions and compulsions appear limited to food and health [15]. People diagnosed with OCD commonly have good insight into their disordered behaviours [3], whereas individuals exhibiting ON tendencies tend not to recognise their behaviour as disordered, wearing their dietary achievements as a badge of honour [17, 24]. Despite some differences between OCD and ON, there remains evidence that OC symptoms play a role in ON [25].

### Phenomenological overlap of ON and OC symptoms

Given the symptom overlap, recent research has focused on whether ON should be classified as an ED or an obsessive–compulsive or related disorder, with many studies concluding that ON is more closely related to an ED [4]. However, this conclusion may be premature. Early developed ON assessments such as the ORTO-15 focused on eating symptoms, potentially failing to capture OC symptoms. Research investigating the relationship between ON and OC symptoms using the ORTO-15 has found vastly inconsistent results [26]. Despite the authors of the ORTO-15 noting the need for a revision and the inclusion additional items to evaluate OC behaviour [27], the ORTO-15 remains a popular assessment and is the predominant assessment in the field. Other commonly used ON assessments such as the Eating Habits Questionnaire (EHQ) [24] demonstrate stronger relationships between ON symptoms and OC symptoms, than that observed when utilising ORTO-15; demonstrating an increase in ON symptoms as OC symptoms increase [21, 28–31]. The stronger relationships found in studies utilising the EHQ may reflect the fact that the development of the EHQ encapsulated more recent research on ON phenomenology and considered OC symptoms.

In addition to OC symptoms, OC beliefs may also form part of ON phenomenology, with little research investigating the role of OC beliefs in ON to date. OC beliefs encapsulate the underlying dysfunctional beliefs that may develop and maintain OC symptoms. People with OCD can believe they are personally responsible for all consequences of their actions, or inactions, and are intolerant of making mistakes, having perfectionistic beliefs, and believing their intrusive thoughts are overly important [32]. In a study comparing ON symptoms between Italian and Polish participants, Brytek-Matera, Pardini et al. found ON symptoms were associated with OC beliefs as assessed by the Obsessive Beliefs Questionnaire (OBQ) [30, 33]. However, the OBQ had not been validated in Polish samples and thus the findings of this study should be interpreted with caution. Although no further identified studies have examined the relationship between ON symptoms and OC beliefs, several studies have investigated the role of perfectionism in ON symptoms.

### Perfectionism as a potential underlying construct

Perfectionism is an OCD-related belief and a common underlying transdiagnostic factor related to eating disorders and other mental illnesses (e.g., depression) [34]. Perfectionism, a broadly defined concept that has been widely explored in the literature, has been theorised as a multidimensional personality trait with two primary domains, perfectionistic strivings (striving for extremely high standards) and perfectionistic concerns (concern about others’ or one’s own evaluation of performance), in addition to alternatively being described as a unidimensional construct termed clinical perfectionism [35].

The multidimensional perfectionism model encapsulates both adaptive and maladaptive perfectionism. Adaptive perfectionism can be beneficial and healthy; for example, an individual with adaptive perfectionistic concerns and strivings may possess the drive to strive towards and meet goals and flexibly adjust their own realistic standards [36]. However, maladaptive perfectionism has been implicated in stress, anxiety, depression, and negative affect, and may negatively impact mental health treatment interventions [37]. For example, individuals may perceive the clinician as critical, exacerbating perfectionistic concerns about their abilities, or individuals may avoid set tasks from the intervention if they feel unable to complete the task perfectly. It may only be clinically relevant to study and treat maladaptive aspects of perfectionism [38].

Clinical perfectionism is a system of self-evaluation in which self-worth is based on one’s ability to successfully meet self-imposed standards despite adverse consequences such as social isolation, insomnia, disordered eating, and depression [5]. Clinical perfectionism is conceptualised as an intrinsically motivated, maladaptive, and pathological type of perfectionism which unlike multidimensional perfectionism does not include any adaptive components [5]. Further, clinical perfectionism can have detrimental impacts on treatment engagement, being implicated in poorer treatment outcomes in depression, bulimia nervosa, and anorexia nervosa, due to relentless determination to excessive standards, and self-evaluation being excessively dependent on achieving those standards [36]. Assessing whether clinical perfectionism plays a role in ON may be more clinically and empirically relevant than assessing both adaptive and maladaptive types of perfectionism together, with potential implications for targeted interventions.

Perfectionism, in general, has been linked to EDs and OCD [34, 39], and has been found to correlate with ON symptoms [12, 28, 40]. However, few studies have investigated the relationship between clinical perfectionism and ON. Yet, Yung and Tabri found a positive indirect relationship between clinical perfectionism and ON symptoms in participants demonstrating erroneous beliefs about eating safely and effectively [41]. Further, Pollack and Forbush found that perfectionism combined with neuroticism moderated the relationship between OC symptoms and body dissatisfaction [20]. It is therefore possible that higher levels of self-criticism and evaluation in clinical perfectionism combined with higher levels of rigidity that accompanies OC symptoms may be associated with ON-type symptoms, particularly given the adherence to specific, self-focused rules found in all three symptom clusters. Conversely, lower levels of clinical perfectionism and OC symptoms may not be associated with ON-type symptoms. More research on the role of clinical perfectionism in ON may develop understanding of underlying and transdiagnostic aspects of ON.

### The current study

This study aimed to investigate if OC symptoms, including OC beliefs, is associated with ON symptomology. In addition, this study aimed to determine if perfectionism moderated the relationship between ON and OC symptoms. It was hypothesised that:

- Higher OC symptoms and OC beliefs would be associated with higher ON symptoms; and
- Perfectionism would moderate the relationship between ON symptoms and OC symptoms, where higher perfectionism levels and higher OC symptoms together would be associated with higher ON symptoms.

## Methods

### Participants

The current study comprised 190 Australian-based participants aged 18–63 (*M* = 28.63, *SD* = 9.88) and predominantly female (80.0% female; 18.9% male; 1.1% other gender). Participants were recruited as part of a larger study using convenience sampling through the Swinburne University of Technology research experience programme, an existing participant registry, and adverts posted in psychology clinics, mental illness advocacy organisations and on social media.

Participants were eligible to participate if 18 years or older and fluent in English. As part of the larger study, exclusion criteria included a history of a head injury involving a loss of consciousness for more than 30 s, any neurological condition (including epilepsy), a history of psychosis or psychotic symptoms, and family history of a psychotic disorder.

### Materials

#### Demographics and screening

Screening and demographic information captured included age, gender, marital status, occupation, employment status, language(s) spoken, country of residence, highest education level attained, and mental health diagnoses. Participants not passing screening, based on inclusion criteria, did not proceed further (*n* = 36).

#### Eating Habits Questionnaire (EHQ) [24]

The EHQ is a 21-item self-report questionnaire assessing ON symptoms on three subscales: problems associated with, knowledge of, and feelings about healthy eating. Items were rated on a 4-point Likert-type scale ranging from 1 to 4 with higher scores indicating more ON symptoms. The EHQ has established validity and demonstrated good internal consistency (*α* = 0.88) in a sample of English speaking university students [42]. The current study’s Cronbach’s alpha was 0.93.

#### Obsessive–Compulsive Inventory Revised (OCI-R) [43]

The OCI-R is an 18-item self-report questionnaire assessing OC symptoms on six subscales: washing, obsessing, hoarding, checking, ordering, and mental neutralising. Items are measured on a 5-point Likert-type scale ranging from 0 to 4, with higher scores indicating more OC symptoms. The OCI-R has demonstrated good to excellent test–retest reliability and internal consistency in samples of healthy controls (total score *α* = 0.81–0.93) [43]. The current study Cronbach’s alpha was 0.92.

#### Obsessive Beliefs Questionnaire (OBQ-20) [44]

The OBQ-20 is a 20-item self-report questionnaire used to assess OC beliefs on four subscales: threat estimation, inflated responsibility for harm, importance and control of thoughts, and perfectionism and intolerance of uncertainty. Responses were measured on a 7-point Likert-type scale ranging from 1 to 7, with higher scores indicating more obsessive beliefs. The OBQ-20 has demonstrated excellent internal consistency (*α* = 0.80–0.82) in an Australian sample [44]. The current study found Cronbach’s alphas of 0.83 to 0.94.

#### Clinical Perfectionism Questionnaire (CPQ) [45]

The CPQ is a 12-item self-report questionnaire measuring clinical perfectionism outside of eating, weight, and appearance, assessing the extent individuals strive to meet high standards, and how such striving impacts self-evaluation when self-imposed standards are not met, which may help distinguish the impact of perfectionism from disordered eating [46]. Items referred to the participant’s experience over the past month and responses were measured on a 4-point Likert-type scale ranging from 1 to 4 with higher scores indicating more clinical perfectionism. The CPQ has demonstrated good validity and acceptable internal consistency in community samples (*α* = 0.71 and *α* = 0.73) [46]. Cronbach’s alpha for the current study was 0.81.

### Procedure

Data collection commenced on May 12, 2022 and concluded on August 8, 2022. Potential participants were provided with a link to the self-report online study which included the assessments described above as part of a larger battery of questionnaires. The study included an explanatory statement before consent was obtained followed by screening questions where ineligible participants did not proceed. The study took approximately 40 min to complete. Other than university students receiving a course credit for their participation, no incentives were offered to participants. This study was approved by the Swinburne University Human Research Ethics Committee (Ref. 20221536-9804) in line with the Australian National Statement on Ethical Conduct in Human Research.

### Design

The current study used an observational, cross-sectional design. An a priori power analysis using G*Power, assuming 80% power estimated a minimum of 124 participants was required to detect a small to medium main effect size in a linear multiple regression controlling for two variables (*f*^2^ = 0.08) [47]. It was accepted that a moderation analysis may impact the ability to detect smaller effects given the complexity of the analysis. However, there was insufficient literature in this domain to estimate a clinically meaningful effect size and thus conduct an a priori power analysis for the moderation analysis.

### Statistical analysis

All analyses were conducted with SPSS Statistics, Version 28.0 [48], two-tailed, with alpha set to 0.05. Bivariate correlation analyses were conducted using Kendall’s tau to determine relationships between ON, OC, and perfectionism symptoms as the assumption of normality was not met.

Prior to conducting a hierarchical linear multiple regression analysis, relevant assumptions were tested. Two univariate outliers and four multivariate outliers were identified but not deemed influential and thus remained in the sample [49]. Data were deemed to be additive and linear, homoscedastic, and residuals were normally distributed. The assumption of multicollinearity was violated and therefore mean-centred perfectionism and OC variables were used in the analysis. The assumption of independence of errors was met. A four-step hierarchical linear regression was conducted to examine the relationship between OC and ON symptoms, and to determine whether perfectionism moderated the relationship between ON and OC symptoms. As some studies reported significant effects of age and gender on ON symptoms, where being younger [50, 51] and female [51, 52] was associated with higher levels of ON symptoms, age and gender were controlled for at step one. OC variables were entered at step two and perfectionism at step three. The interaction term (OC symptoms and perfectionism) was entered at step four and the significant interaction was analysed using simple slopes analysis. A follow-up simple slopes analysis was conducted using Hayes’ PROCESS macro for SPSS version 4.1 [53] to determine if the moderation effect was significant.

## Results

### Preliminary data screening

Of 244 recruited participants, 36 did not meet screening criteria. Additional participants were removed as they did not consent to participate (*n* = 1), did not proceed after providing consent (*n* = 3), failed seriousness checks (*n* = 6; a question checking attention and random responding, time elapsed completing the study, and invalid response patterns), or recorded more than 30% missing values on an assessment (*n* = 8).

After the exclusion of the above-described participants, it was noted that across the entire data set, eight values (< 0.06%) were missing, all on the EHQ assessment. Little’s Missing Completely at Random (MCAR) test indicated values on the EHQ Problems subscale were not MCAR, χ^2^ (33, *N* = 190) = 65.32, *p* < 0.001. However, as so few values were missing, values were imputed using SPSS expectation–maximisation [54]. Analyses were repeated with and without affected participants and there was minimal difference in results. Therefore, the estimated level of bias introduced by expectation–maximisation was considered negligible. The dataset with the imputed values was used for all analyses presented in this paper.

### Descriptive statistics

Demographic characteristics of the sample are presented in Table 1.

**Table 1:** Sample demographic characteristics

Demographic variable	*n*	%
Gender		
Female	152	80.0
Male	36	18.9
Other	2	1.1
Current employment status		
Student	66	34.7
Employed	116	61.0
Home duties	3	1.6
Unable to work due to illness	4	2.1
Unemployed	1	0.5
Self-reported lifetime mental illness		
Anxiety disorder	37	19.5
Eating disorder	31	16.3
Mood disorder	36	18.9
Obsessive–compulsive disorder	6	3.2

### Correlations

Statistically significant positive moderate relationships were observed between the EHQ and OCI-R, OBQ-20, and CPQ, with higher ON tendencies associated with higher OC and perfectionism symptoms. Table 2 summarises mean scores observed, Cronbach’s alpha values and Kendall’s tau correlations between scales and subscales. OC symptoms and beliefs were associated with ON problems subscale and OC ordering and perfectionism was associated with all ON subscales.

**Table 2:** Descriptive statistics and Kendall’s tau correlation coefficients for ON, OC and perfectionism symptoms

Scale	*M*	*SD*	*α*	Correlations			
				EHQ-Total	EHQ-Problems	EHQ-Knowledge	EHQ-Feelings
EHQ total	39.74	11.82	0.93	–	–	–	–
Problems	19.16	7.72	0.93	0.75***	–	–	–
Knowledge	9.92	3.22	0.77	0.72***	0.49***	–	–
Feelings	10.67	2.77	0.77	0.61***	0.37***	0.52***	–
OCI-R total	19.52	13.17	0.92	0.24***	0.31***	0.13*	0.12*
Obsessing	4.42	3.55	0.91	0.22***	0.27***	0.10	0.14*
Hoarding	3.75	2.76	0.74	0.15**	0.25***	0.01	0.06
Ordering	4.21	3.04	0.88	0.24***	0.26***	0.20***	0.15**
Checking	2.93	2.62	0.77	0.12*	0.19***	0.04	0.04
Neutralising	2.07	2.67	0.77	0.16**	0.22***	0.11*	0.04
OBQ-20 total	72.75	24.21	0.94	0.23***	0.31***	0.10	0.09
Threat	17.49	7.16	0.86	0.19***	0.27***	0.04	0.06
Responsibility	20.82	6.68	0.83	0.18***	0.22***	0.11*	0.10
Thoughts	15.00	7.20	0.86	0.21***	0.30***	0.09	0.06
Perfectionism	19.44	7.68	0.87	0.22***	0.26***	0.14**	0.12*
CPQ total	29.12	6.16	0.81	0.31***	0.29***	0.22***	0.24***

*N* = 190

*p < 0.05

**p < 0.01

***p < 0.001

### Hierarchical linear regression

The hierarchical linear regression revealed that at step one, age and gender significantly contributed to the regression model, *F*(2, 187) = 4.61, *p* = 0.01, accounting for 4.7% of the variation in ON symptoms. After controlling for age and gender, and to test the first hypothesis, an additional 22.9% of variation in ON symptoms was explained by mean-centred OC symptoms and OC belief variables entered at step 2. This change in *R*^2^ was significant, Δ*F*(2, 185) = 29.29, *p* < 0.001, with medium effect size (*f*^2^ = 0.38). Together, the four predictor variables explained 27.6% of ON symptom variance, *R*^2^ = 0.28, adjusted *R*^2^ = 0.26, *F*(4, 185) = 17.64, *p* < 0.001.

Adding perfectionism to the regression model at step 3 significantly explained an additional 9.4% of the variation in ON symptoms, Δ*F*(1, 184) = 27.36, *p* < 0.001. The combined five variables explained 37.0% of the variation in ON symptoms, *R*^2^ = 0.37, adjusted *R*^2^ = 0.35, *F*(5, 184) = 21.60, *p* < 0.001. After perfectionism was entered into the model, OC beliefs were no longer significantly associated with ON symptoms. Finally, the addition of the interaction term to the regression model to test the second hypothesis significantly explained an additional 2.2% of the variation in ON symptoms, Δ*F*(1, 183) = 6.47, *p* = 0.01, *f*^2^ = 0.03. Perfectionism was the most important predictor of ON symptoms, uniquely explaining 8.3% of the variation in ON symptoms. Together, all six variables accounted for 39.1% of ON symptom variance, *R*^2^ = 0.39, adjusted *R*^2^ = 0.37, *F*(6, 183) = 19.61, *p* < 0.001, *f*^2^ = 0.64. Table 3 presents the regression coefficients for the hierarchical linear regression analysis.

**Table 3:** Summary of hierarchical linear regression analysis for OC tendencies and perfectionism predicting ON symptoms

Variable	*B*	*SE B*	95% CI for *B*	β	*sr*^*2*^
Step 1					
Constant	31.43***	3.17	25.17, 37.69		
Gender	1.14	2.06	− 2.91, 5.20	0.04	0.002
Age	0.26**	0.09	0.09, 0.43	0.22	0.05
Step 2					
Constant	24.34***	4.03	16.39, 32.30		
Gender	− 2.27	1.86	− 5.93, 1.40	− 0.08	0.01
Age	0.27***	0.08	0.13, 0.42	0.23	0.05
OC symptoms	0.24**	0.08	0.09, 0.40	0.27	0.04
OC beliefs	0.13**	0.04	0.05, 0.21	0.27	0.04
Step 3					
Constant	33.86***	4.19	25.60, 42.12		
Gender	− 2.68	1.74	− 6.11, 0.75	− 0.09	0.01
Age	0.30***	0.07	0.16, 0.44	0.25	0.06
OC symptoms	0.24**	0.07	0.09, 0.38	0.26	0.04
OC beliefs	− 0.01	0.05	− 0.10, 0.09	− 0.01	< 0.001
Perfectionism	0.81***	0.15	0.50, 1.11	0.42	0.09
Step 4					
Constant	33.69***	4.13	25.54, 41.83		
Gender	− 2.89	1.72	− 6.27, 0.50	− .10	0.01
Age	0.29***	0.07	0.15, 0.43	0.24	0.06
OC symptoms	0.21**	0.07	0.07, 0.36	0.24	0.03
OC beliefs	− 0.01	0.05	− 0.10, 0.08	− 0.02	< 0.001
Perfectionism	0.76***	0.15	0.46, 1.07	.40	0.08
OC symptoms × Perfectionism	0.02*	0.01	0.01, 0.04	0.16	0.02

*CI* confidence interval

Gender coded as 0 = Male, 1 = Female, 2 = Other; independent variables (except age and gender) centred to mean; *N* = 190; *R*^2^ = 0.05 for Step 1, ∆*R*^2^ = 0.23 for Step 2, ∆*R*^2^ = 0.09 for Step 3, ∆*R*^2^ = 0.02 for Step 4

*p < 0.05

**p < 0.01

***p < 0.001

**Fig. 1:**
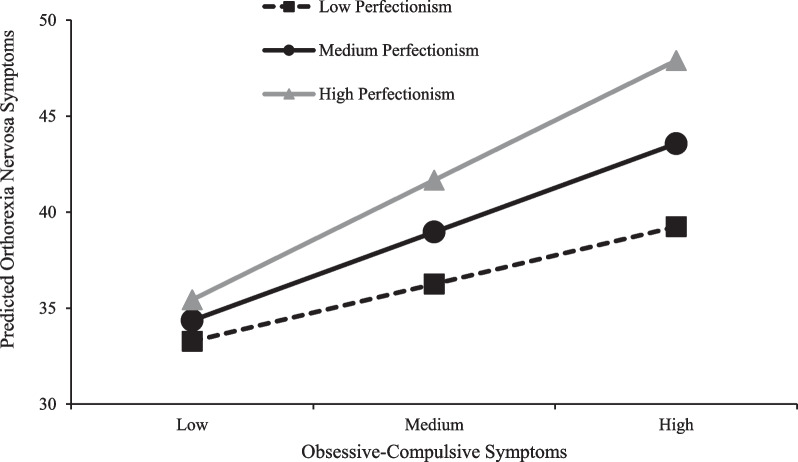
Interaction effect of OC symptoms and perfectionism symptoms on predicted ON symptom scores. Independent variables centred to mean

**Fig. 2:**
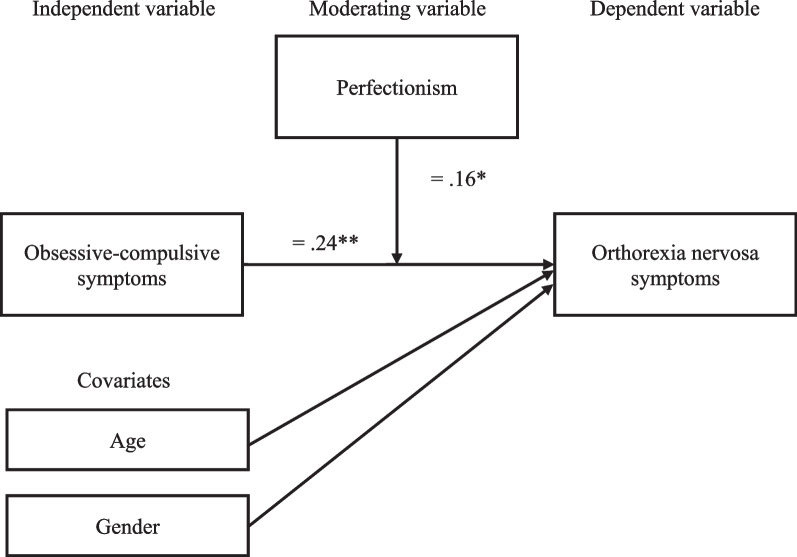
Model of study findings. *β* = Based on mean-centred variables; *p < 0.05, **p < 0.01

The results revealed a significant moderating effect of perfectionism on the relationship between ON and OC symptoms (*b* = 0.02, *t* = 2.54, *p* = 0.01). As shown in Table 4, OC symptoms were significantly related to ON symptoms when perfectionism was at the mean score (*p* = 0.004) and one standard deviation above the mean score (*p* < 0.001), but not when perfectionism was one standard deviation below the mean score (*p* = 0.35). Figure 1 demonstrates a simple slopes analysis at low, medium, and high levels of OC symptoms for participants with low, medium, and high levels of perfectionism (at mean and one standard deviation above and below the mean score). Results demonstrate that participants with high levels of perfectionism have higher levels of ON symptoms than those with medium levels of perfectionism who, in turn, have higher level of ON symptoms than those with low levels of perfectionism at low, medium, and high levels of OC symptoms. These differences in ON levels increase in all three levels of perfectionism as the levels of OC symptoms increases. Figure 2 demonstrates a model of study findings.

**Table 4:** Conditional effects of OC on ON symptoms

Perfectionism	*β*	*p*	95% CI for *B*
One *SD* below mean	0.08	0.35	− 0.09, 0.25
At the mean	0.21	0.004	0.07, 0.34
One *SD* above the mean	0.33	< 0.001	0.17, 0.49

*SD* standard deviation

## Discussion

This study aimed to determine if OC symptoms and beliefs were associated with ON symptoms and if the relationship was moderated by perfectionism. As hypothesised, higher OC tendencies were associated with higher ON symptoms and the relationship between OC symptoms after controlling for age and gender, and ON symptoms was moderated by perfectionism.

### ON/OC symptom relationship

Results from the current study highlight the larger role that OC symptoms may have in ON symptoms than previously reported in the literature [4]. The greater effect size found in the current study may reflect the broader spectrum of OC concerns in the current general population sample relative to targeted sample of healthcare and non-healthcare professionals [55], or dieticians [56], or a broader assessment of symptoms with the EHQ relative to the ORTO-15 in previous studies [12, 56]. Relationship findings are consistent with previous studies using the EHQ, which have demonstrated moderate effect sizes [21, 28–31]. The consistently supported moderate ON/OC relationship throughout studies which have used the EHQ provides further evidence for the potential measurement issues of the ORTO-15, which may underestimate the role of OC symptoms in ON phenomenology [22, 52, 57–61].

ON symptom total scores in the current study were significantly correlated with all OCI-R and OBQ-20 subscales, consistent with Brytek-Matera, Pardini et al. who similarly found significant associations [30]. However, in the current study, OC belief significance may have been explained predominantly by perfectionism, given none of the beliefs were significantly associated with ON symptoms after introducing clinical perfectionism scores into the regression model. Aside from perfectionism/intolerance for uncertainty, most of the OC beliefs captured by the OBQ-20 (e.g., importance of thoughts/overestimation of threat) are focused on the harm-avoidance aspects of OC symptomology [44]. Some OC beliefs such as importance and control of thoughts may have less relevance to the obsessional aspects in ON.

### Perfectionism as a moderator of the ON/OC relationship

Perfectionism was found to play a moderating role in the relationship between OC and ON symptoms with a small effect size. Although no other known study has investigated the moderating role of perfectionism in ON symptoms, results are consistent with studies investigating perfectionism in ON phenomenology reporting moderate correlations [12, 40] even after controlling for OC symptoms [28]. However, these studies investigated multidimensional perfectionism that encapsulates broader perfectionism concepts, including adaptive and external motivations, rather than clinical perfectionism that focuses on the dysfunctional, intrinsically motivated concept of perfectionism. Consistent with current results, Yung and Tabri found clinical perfectionism was directly and indirectly associated with ON symptoms via defining oneself with an inflated focus on health [41]. Yung and Tabri’s results evaluating clinical perfectionism outside of the boundaries of eating, weight, and appearance suggest perfectionism may extend to multiple domains in an individual’s life, presenting as a vulnerability to becoming obsessed and fixated in various manifestations. In the case of ON, this fixation could be centred around perceived healthy eating. Perfectionism appears to consistently demonstrate a role in ON phenomenology, with early indications clinical perfectionism may be a significant factor.

### Implications of study findings

The limited OC beliefs component in ON in regression results could be considered consistent with differences between ON and OCD. Whereas individuals with OCD have ego-dystonic thoughts needing to be controlled, individuals with ON appear to be ego-syntonic, where thoughts may not be considered threatening or important to control, and restricting diet may ease distress and anxiety through exerting control over their environment [15]. So long as individuals with ON adhere to their “healthy” diet, they avoid the threat of feared health consequences (i.e. harm avoidance). Although current study results indicate a limited role for OC beliefs other than perfectionism in ON phenomenology, further empirical research in this area would facilitate further understanding of the role, if any, of the other OC beliefs such as the overvalued importance of their intrusive thoughts [32].

If perfectionism moderates the ON/OC symptom relationship as current study results suggest, it may help explain the inconsistent findings of the relationship between OC and ON symptoms in the literature. If OC symptoms are only apparent alongside high levels of perfectionism, results reporting the relationship between ON symptoms and OC symptoms may reflect the level of clinical perfectionism in the samples studied rather than levels of OC symptoms alone. As such, the current study results may reshape how OC symptoms are considered in ON phenomenology.

As perfectionism itself is quite prevalent in ON [12, 28, 40], perfectionism may be driving obsessions about food and health in addition to other OC symptoms. ON may be an expression of being perfectionistic about health or avoiding disease, with some evidence suggesting health anxiety may play a role in ON phenomenology [62]. Perfectionistic rigidity may explain the OC component to ON more accurately than OC symptoms alone, especially considering the considerable symptom overlap between ON and perfectionism. Further research is needed to delve deeper into how perfectionism presents in ON, with self-efficacy and self-esteem being encouraging areas of research on perfectionism and bulimia nervosa [63, 64].

Perfectionism appears to be an important consideration for treatment of ON [34]. Future research might distinguish between the different aspects of perfectionism that can be targeted for ON treatment, such as perfectionistic attitudes, beliefs, and/or self-talk [36]. Further, as the clinical perfectionism assessment used in the current study encapsulates self-evaluation and self-worth [5], interventions investigating how striving and achievement is entangled with self-identity might be an avenue for future ON intervention exploration. Indeed, current cognitive-behavioural interventions would benefit from drawing attention to and challenging the high self-imposed standards and self-evaluation seen in clinical perfectionism with graded exposure in lowering standards [5].

### Limitations and future research

The current study was not without limitations. Beyond the correlational nature of the study being unable to determine causality, sample size limited power to conduct more detailed analyses and investigate alternative explanations for results. For example, perfectionism is quite broad and has been implicated widely in psychopathology. Future research might explore more detailed aspects of perfectionism to target certain perfectionistic behaviours for interventions. Moderation analyses typically recruit much larger sample sizes to be able to report stable estimates [65]. Moderation results in small samples may overestimate the true effect size and thus our findings should be interpreted with caution.

The current study was also limited by only studying perfectionism as a potential moderator when, for example, both perfectionism and neuroticism have been found to moderate the relationship between OC symptoms and body dissatisfaction [20]. Future research could benefit from teasing apart various cognitive factors and how they interact in ON symptomology.

### Conclusion

OC beliefs do not appear to play a large role in ON symptomology, but there is evidence to suggest OC symptoms are moderately common. The interaction of perfectionism and OC symptoms may drive obsessions about food and health in ON rather than broader OC symptoms. This research has important implications for both further defining ON phenomenology and developing effective treatments for ON.

### What is already known on this subject?

Orthorexia nervosa (ON), a proposed disorder describing an obsessive focus on “healthy” eating, is characterised as having overlapping symptoms with obsessive–compulsive disorder. However, ON/obsessive–compulsive (OC) symptom relationships are inconsistently reported in the literature. Perfectionism has been linked to eating disorders, including ON, and obsessive–compulsive disorder. Therefore, levels of perfectionism may explain some of the variability in the relationship between ON and OC symptoms.

### What this study adds

Obsessive–compulsive symptoms appear to be more common in orthorexia nervosa than indicated in previous literature. Additionally, perfectionism may moderate the relationship between obsessive–compulsive symptoms and orthorexia nervosa symptoms, whereby higher levels of obsessive–compulsive symptoms were associated with higher levels of orthorexia nervosa but only with higher levels of perfectionism. Perfectionism, rather than obsessive–compulsive symptoms, may be driving food and health obsessions in orthorexia nervosa which may be helpful when developing and delivering treatment.

## Data Availability

The data that support the findings of this paper are not available due to ethical constraints and in accordance with participant confidentiality and informed consent.
